# Plastic Fusible Metal for Filling Teeth

**Published:** 1863-09

**Authors:** B. Wood


### PLASTIC FUSIBLE METAL FOR FILLING TEETH.

BY B. WOOD, M. D.

[Read before the American Dental Convention, Saratoga Springs, Aug. 1863.]

Gentlemen of the Convention:—Allow me to call your attention to the improved form of the new fusible metal discovered and patented some time since, as now applied and used for the purpose of filling teeth. It is designed as a substitute for gold foil, where economy is an object, as well as for the inferior materials. For this it is believed to supply a very important desideratum long felt to exist by the dental profession and the community. It forms solid and impervious fillings which so perfectly adapt themselves on introduction to the sinuosities and walls of dental cavities as to preclude the admission of air or moisture at every point. At a moderate heat, but little higher than that at which hot drinks are taken into the mouth, and yet not low enough to be the least affected by them, it is rendered soft and plastic, and can be moulded into any shaped cavity, or built out from defective teeth, restoring their form and usefulness. Upon introduction into a tooth it becomes at once hard and solid, and therefore is fit for immediate use in mastication, and not subject, like amalgam or "cement" plugs, to rub off by the attrition of food or to crumble out. It contains no mercury, nor is it allied in composition or qualities to amalgams ; and it is exempt from the important objections urged against these preparations. Although liable to cloud upon the surface in some mouths, this is but slight; generally little more than a dull, opaque appearance, hardly amounting to a perceptible change of color. In no case have I found it ever to blacken on the surface as amalgam and also silver plate usually do; and it never discolors the tooth or contiguous portions of the dentine as amalgam so uniformly does. Being much harder than solid block-tin, or nearly as hard as pure gold, it will outwear any tin plug, however perfectly consolidated, as well as plugs of gold foil imperfectly condensed; while it requires no force of pressure for its introduction.

These qualities render this material an object of no slight interest to dentists, as well as to those of the community who have defective teeth which they wish preserved in the most effectual as well as the most economical manner. I would respectfully refer those who desire to acquaint themselves fully in regard to the history and nature of this discovery and kindred subjects to the scientific and professional journals, only designing to draw attention here to the value of the material applied and adapted to the purpose of filling teeth; referring in particular to the following sources of information: U. S. Mining Journal, May 26th, 1860; Silliman's American Journal of Science and Arts, Sept. 1860, and March 1862; Scientific American, Nov. 17th, 1860, and April 5th, 1862; Journal of Franklin Institute, Aug. 1860, and Jan. 1861 and 1862; or the Annual of Scientific Discovery for 1861, &c. See also the Dental Journals, 1860–1863, *passim.*

In the Dental Register of the West for 1862 and 1863 may be found a series of papers on "Metals and Alloys," in which, among others, the component metals of this material are particularly described. The chief design of these papers was to give the results of actual experiment, omitting, except so far as was necessary for connection and a certain degree of completeness, such matter as was accessible from the ordinary sources. Although written under disadvantages, and necessarily incomplete in themselves, it is believed that, in connection with what may be gleaned from the books, they will add no little to the common stock of information in regard to the nature and mutual relations of a class of elements which appear to have been the most neglected in the very particulars which give them the greatest practical interest.

I am aware that some undervalue metallurgic study and research as not practical. But even if it were not, ought it not be held in some repute for its own sake as an important branch of science by a scientific pıofession? Nor is it without practical bearing. No branch is more practical to *all* the arts, and especially our own. Those who would ignore it as of slight valuation, can do so only because they have yet to learn the *alphabet* of the science upon which they assume to pronounce judgment. And I will venture that professional teachers who treat this branch with apathy or derision, will prove, upon critical inspection, to be not the most trustworthy in other branches. Its practical importance could be illustrated by a thousand instances coming home to us daily. But this would be quite too wide from the object of this paper, which is only to draw attention to one of the additions which metallurgy has made to the resources of practical dentistry.

If the discovery of the new fusible metal was sufficient to interest the scientific world, its successful adaptation and application to the purpose of filling teeth ought surely to claim some interest on the part of dentists. Let it not be despised because brought forward by a *dentist.* The scientific world, though long led to despise our fraternity as much as we have despised each other, are beginning to recognize that some good can come out of Nazareth, and to award due credit for it, despite their prejudice; and but for the low jealousy still too prevalent among us, which grudges a just award to our professional brethren, we should soon win that respect to which the profession is justly entitled. But, if to be honored one must honor his own household, it becomes us as a body to receive with consideration whatever comes from a professional brother, *because of its source*, until it shall prove unworthy of that source. Let it be no longer said that we evince more confidence and fairness toward offerings from the veriest ignoramuses outside of our own pale than toward those proposed by our own members, even though presented by men whose whole lives in the service of the profession, public and private, afford not a solitary ground for distrust of their integrity, and whose qualifications are well enough known to their compeers, if they had the generosity to avow it.

For what I have to offer, I only ask a fair hearing and fair examination due from man to man. I certainly should not have proposed it if I had not been well assured that it would prove of value. Those who have tested it most thoroughly know that the claims upon which I proposed it fell far short of its merits. Its suitableness and applicability have been demonstrated in cases not at first contemplated, and the field of its usefulness greatly extended. Every dentist who has learned to use it has spoken well of it, so far as I have been able to hear an expression of opinion.

The Dental Register of the West, comparing the Plastic Metallic Filling with tin foil (the best substitute for gold hitherto used), says, Sept. 1862 :—

"We think this alloy quite as good in any respect as tin, and in some, perhaps, much better. In the facility with which it can be introduced and manipulated, it is certainly much superior to tin foil, and then it is in a solid mass when introduced."

In a subsequent number of the same journal (which see for valuable suggestions as to the manner of applying the "Filling," too lengthy for citation here), the editor remarks :—

"It can be used in any place where the tin foil can, and in many cases where it can not. For filling up large cavities in molar teeth where the use of gold is not admissible, it is the best preparation we have. * * * It can rapidly and readily be built into any required shape. We have, in a number of cases, used it for temporary filling, in the treatment of sensitive dentine, and particularly in the treatment of exposed nerves, when the object is to protect an exposed point till new dentine is formed, or until the conditions are so changed as to admit a permanent filling."—(*Dental Register*, Nov. 1862.)

The uniform expression of dental practitioners skilled in the use of the material is to a similar purport, if I felt at liberty to cite them.

But I may be allowed to refer to Prof. Taft, from whose journal I have quoted the above, well known as the author of our standard text-book on "Operative Dentistry," and one of the most accomplished operators with gold foil in the West. Referring to the Plastic Metallic Filling, in a letter dated Oct. 19, 1862, he remarks:—

"I use this far more than I have used anything of the kind for ten years. It will certainly be largely used by those who make cheap fillings."

At a later date, April 1863, Prof. Taft writes:—"It is without all doubt the best thing except gold yet known for filling teeth. I am better pleased with it than ever; many are using it from my suggestion, and all are pleased with it so far as I know."

Dr. Atkinson, of New York city, our pioneer in the higher branches of the profession, theoretically and practically well known for his remarkable success in surgical treatment and for his magnificent fillings with gold, his favorite material, writes April 22, 1863, as follows:—

"I have been using very cautiously for the last year your Plastic Metallic Filling, which I am happy to say suits me next best to gold for preserving decayed teeth. It must come into very general use so soon as known."

Again he says: "I look upon it as a great blessing to the poor classes of those having decayed teeth."

Dr. J. S. Wood, of Lansing, Mich., late of Albany, N. Y. where his reputation as a dental practitioner for twenty-five years, and his great success in the use of this material the past year have so many witnesses, and who, it is due to say, is the inventor of the most useful form of instrument yet constructed for applying the metal, recommends it as "an article vastly superior to anything except gold for filling teeth, and in many cases superior even to gold."

To add anything further from competent judges yet heard from, would be only to reiterate the same facts in nearly the same language; but so far seemed fit to place on record, since each of those referred to has done much to extend or to facilitate the applicability of the material of which they speak.

For my own part although satisfied of its merits before proposing it, I have been content to leave them to be gradually disclosed by others. But I feel that I ought not *withhold* the facts after such corroboration. As an earnest of its capabilities and the estimate set upon it so far as already known to the profession and community, I may mention that competent operators are now *building out entire crowns of teeth* with it; for which I am informed that in the City of New York the best skill commands ten dollars and upwards for each case. An article with which such results are capable of being achieved is surely worth being made known.

In view of objections on the grounds of its being "a patent," so benevolently urged by parties engaged in the manufacture, sale, and use of other materials with which it comes in competition, I beg to say, that although patented (and with a view to some ultimate remuneration, not to mention higer considerations which will appear in the sequel), yet I have not up to the present time required any fee for the right to use it, nor ever charged one dime for instructions. On the contrary, purchasers of the material to an amount sufficient for initatory trial and practice with it—or one ingot of $\frac{3}{4}$ oz. weight and sufficient for 100 plugs, at the cost of $2—have been furnished free of charge with a grant to use it in their office practice, protecting the holder ever after from interference on the part of subsequent licensees. Of course these grants do not confer any exclusive privileges to the detriment of others, nor the right to manufacture and sell the material; that is to say, they benefit only *dentists;* in their ordinary sphere of business, and not manufacturers and dealers.

Although this course is inconvenient to myself and not likely to be taken advantage of by the majority for whose benefit it was adopted, it will still be pursued for the present to afford all to whom it was offered a fair opportunity to profit by it, when resort will be had to the customary plan of issuing regular licenses with a commensurate fee affixed. Indeed, to save myself, I should have to do this or raise the price of the material which in itself will not compensate the time and expense involved. Of the two, the plan proposed has the sanction of custom, and is, undoubtedly, the best for all parties. A reference to the facts will make this appear. Although with a few, ingenuity may supply the want of instruction and of suitable instruments, yet the great majority are unable to succeed unless furnished with these at the start. Many, after all that has been said and written, have but the vaguest idea in regard to the material and the manner of using it. I find the most of those who take hold of it resort to very unsuitable instruments, often the most worthless cast-off tool about their office. Failing under such circumstances, and it is a marvel if they do not, they become discouraged and condemn the material. Others, not realizing that practice makes perfect, if not succeeding in a few indifferent trials throw it one side, or they apply it at an improper temperature and prejudice their patient and themselves the first attempt. A prevalent mistake is that a few operations ought to suffice to acquire its use and demonstrate its utility. Dentists write me requesting samples enough for a few plugs in order to give it a "trial," when, they say, if they find it as recommended they will buy. Those who have learned to work it know very well what would be the result would I comply with all such requests, post-paid. Now the proper remedy for these drawbacks is clearly to fix a license fee that shall cover all costs, and then furnish the material, instruments, and instructions necessary to acquiring success with it, and to prohibit its use to those who will not take the preparatory means of perfecting themselves in its use. This, too, will protect the profession against those who, though naturally more apt to acquire a new art than many confirmed in old modes, yet do not possess other qualifications necessary to the exercise of our profession. It would secure the benefits of the material mostly to competent operators in reputable practice, until the public shall have learned to discriminate between its use and abuse, and to avoid the incompetent.

